# Supplementary material for: Expression of SREBP-1c Requires SREBP-2-mediated Generation of a Sterol Ligand for LXR in Livers of Mice
Source: eLife. 2017 Feb 28;6:e25015. doi: 10.7554/eLife.25015 (PMC5348127; doi:10.7554/eLife.25015)
Supplement: Supplementary file 1. — Male mice (12–13 wks old in study 1 and 16–18 wks old in study 2) fed chow ad lib were sacrificed and liver sterols and oxysterols were measured with mass spectrometry. Each value represents mean ± SEM. * denotes the level of statistical significance of p<0.05 (Student’s t test) between WT and hepatocyte-Srebf-2-/- mice. DOI: http://dx.doi.org/10.7554/eLife.25015.012 [file elife-25015-supp1.docx]

| **Supplementary file 1. Liver sterol and oxysterol concentrations** | | | | |
| --- | --- | --- | --- | --- |
|  | Study 1 | | Study 2 | |
|  | WT (n=6) | *Srebf-2^-/-^* (n=6) | WT (n=5) | *Srebf-2^-/-^* (n=4) |
|  | ng/mg of liver | ng/mg of liver | ng/mg of liver | ng/mg of liver |
| ***Sterols*** |  |  |  |  |
| 7-Dehydrodesmosterol | 0.00 ± 0.00 | 0.34 ± 0.03 * | N.D. | N.D. |
| Zymosterol | 0.10 ± 0.01 | 0.20 ± 0.03 * | 0.07 ± 0.02 | 0.07 ± 0.02 |
| Desmosterol | 1.87 ± 0.17 | 5.37 ± 0.64 ***** | 1.64 ± 0.14 | 2.84 ± 0.39 ***** |
| Cholestenone | 0.05 ± 0.00 | 0.04 ± 0.00 | 0.34 ± 0.03 | 0.26 ± 0.03 |
| 8-Dehydrochol | 0.98 ± 0.07 | 2.59 ± 0.28 * | 0.79 ± 0.09 | 1.05 ± 0.12 |
| 7-Dehydrochol | 1.05 ± 0.08 | 1.19 ± 0.13 * | 1.47 ± 0.07 | 1.74 ± 0.10 |
| 14-Desmethyl lanosterol | 3.51 ± 0.35 | 2.83 ± 0.37 | 1.07 ± 0.08 | 1.30 ± 0.07 |
| Lanosterol | 1.53 ± 0.19 | 1.93 ± 0.29 | N.D. | N.D. |
| Cholestanol | 9.02 ± 0.72 | 4.00 ± 0.41 | N.D. | N.D. |
| Stigmasterol | 12.20 ± 1.57 | 7.00 ± 1.18 * | 1.61 ± 0.13 | 1.49 ± 0.10 |
| Sitosterol | 25.82 ± 3.05 | 17.66 ± 4.87 | 16.19 ± 0.69 | 19.07 ± 1.19 |
| 24-dihydro lanosterol | 17.92 ± 1.70 | 26.40 ± 2.25 * | 0.21 ± 0.04 | 0.18 ± 0.04 |
| ***Oxysterols*** |  |  |  |  |
| 22OH-Cholesterol | N.D. | N.D. | 0.01 ± 0.00 | 0.00 ± 0.00 |
| 25OH-Cholesterol | 0.01 ± 0.00 | 0.01 ± 0.00 | 0.01 ± 0.00 | 0.01 ± 0.00 |
| 24OH-Cholesterol | 0.12 ± 0.02 | 0.14 ± 0.05 | 0.07 ± 0.00 | 0.06 ± 0.01 |
| 24OXO-Cholesterol | 0.22 ± 0.03 | 0.36 ± 0.08 | 0.07 ± 0.01 | 0.05 ± 0.01 |
| 27OH-Cholesterol | 0.25 ± 0.02 | 0.16 ± 0.03 * | 0.22 ± 0.02 | 0.21 ± 0.04 |
| 24,25 EC-Cholesterol | 0.04 ± 0.01 | 0.29 ± 0.05 * | 0.05 ± 0.02 | 0.05 ± 0.01 |
| 7αOHC-Cholesterol | 0.20 ± 0.03 | 0.12 ± 0.02 * | 0.19 ± 0.01 | 0.20 ± 0.03 |
| 7OXO-Cholesterol | N.D. | N.D. | 0.69 ± 0.01 | 0.46 ± 0.14 |
| 6α-Cholesterol | 0.10 ± 0.02 | 0.00 ± 0.00 * | 0.07 ± 0.00 | 0.05 ± 0.01 |
| 6OXO-Cholesterol | N.D. | N.D. | 0.02 ± 0.00 | 0.01 ± 0.00 |
| 19OHC-Cholesterol | N.D. | N.D. | 0.29 ± 0.06 | 0.24 ± 0.02 |
| 5αOHC-Cholesterol | N.D. | N.D. | 0.12 ± 0.02 | 0.10 ± 0.01 |
| 5,6βOHC-Cholesterol | 0.24 ± 0.00 | 0.21 ± 0.01 | 0.47 ± 0.05 | 0.47 ± 0.06 |
| 5,6αOHC-Cholesterol | 0.08 ± 0.00 | 0.06 ± 0.00 | 0.17 ± 0.02 | 0.17 ± 0.020 |
| 4βOH-Cholesterol | 0.80 ± 0.12 | 0.22 ± 0.02 * | 0.17 ± 0.02 | 0.17 ± 0.02 |

Male mice (12-13 wks old in study 1 and 16-18 wks old in study 2) fed chow *ad lib* were sacrificed and liver sterols and oxysterols were measured with mass spectrometry. Each value represents mean ± SEM. * denotes the level of statistical significance of *P*<0.05 (Student’s *t* test) between WT and hepatocyte*-Srebf-2^-/-^* mice.
