# Supplementary material for: Expression of SREBP-1c Requires SREBP-2-mediated Generation of a Sterol Ligand for LXR in Livers of Mice
Source: eLife. 2017 Feb 28;6:e25015. doi: 10.7554/eLife.25015 (PMC5348127; doi:10.7554/eLife.25015)
Supplement: Supplementary file 2. — Liver was snap frozen and mRNA prepared from the mice shown in Table 1. Each value represents mean ± SEM. * denotes the level of statistical significance of p<0.05 (Student’s t test) between WT and hepatocyte-Srebf-2-/- mice. DOI: http://dx.doi.org/10.7554/eLife.25015.013 [file elife-25015-supp2.docx]

| **Supplementary file 2. Relative mRNA expression in livers of WT and hepatocyte*-Srebf-2^-/-^* mice of Table 1** | | |
| --- | --- | --- |
|  | WT | *Srebf-2^-/-^* |
| ***SREBP Pathway*** |  |  |
| SREBP-2 | 1.0 ± 0.1 | 0.3 ± 0.0* |
| SREBP-1a | 1.0 ± 0.0 | 0.8 ± 0.1 |
| SREBP-1c | 1.0 ± 0.2 | 0.1 ± 0.0* |
| S1P | 1.0 ± 0.0 | 1.2 ± 0.1* |
| Scap | 1.0 ± 0.0 | 0.8 ± 0.0* |
| Insig-1 | 1.0 ± 0.1 | 0.5 ± 0.0* |
| ***Cholesterol Metabolism*** |  |  |
| HMG-CoA synthase | 1.0 ± 0.3 | 0.4 ± 0.1* |
| HMG-CoA reductase | 1.0 ± 0.1 | 0.6 ± 0.1* |
| Farnesyl diphosphate synthase | 1.0 ± 0.1 | 0.4 ± 0.0* |
| Squalene synthase | 1.0 ± 0.1 | 0.5 ± 0.0* |
| LDLR | 1.0 ± 0.1 | 0.8 ± 0.1 |
| PCSK9 | 1.0 ± 0.1 | 0.4 ± 0.0* |
| ***Fatty Acid Metabolism*** |  |  |
| ATP citrate lyase | 1.0 ± 0.1 | 1.0 ± 0.1 |
| ACC1 | 1.0 ± 0.1 | 0.5 ± 0.1* |
| ACC2 | 1.0 ± 0.2 | 0.7 ± 0.1 |
| Fatty acid synthase | 1.0 ± 0.1 | 0.3 ± 0.0* |
| ELOVL6 | 1.0 ± 0.1 | 0.5 ± 0.0* |
| SCD 1 | 1.0 ± 0.2 | 0.0 ± 0.0* |
| ***LXR and LXR-regulated Genes*** |  |  |
| LXRα | 1.0 ± 0.1 | 0.9 ± 0.1 |
| LXRβ | 1.0 ± 0.1 | 1.0 ± 0.1 |
| ABCG5 | 1.0 ± 0.1 | 0.3 ± 0.1* |
| ABCG8 | 1.0 ± 0.1 | 0.4 ± 0.1* |
| ***Control Gene*** |  |  |
| Apo B | 1.0 ± 0.1 | 1.0 ± 0.1 |
|  |  |  |

Liver was snap frozen and mRNA prepared from the mice shown in Table 1. Each value represents mean ± SEM. * denotes the level of statistical significance of *P*<0.05 (Student’s *t* test) between WT and hepatocyte*-Srebf-2^-/-^* mice.
